# How credible is REACH regulation without transparency, quality criteria, assurance, and control?

**DOI:** 10.12688/openreseurope.19510.1

**Published:** 2025-04-10

**Authors:** Antti Joonas Koivisto, Michael Jayjock

**Affiliations:** 1 ARCHE Consulting, Wondelgem, B-9032, Belgium; 2 Institute for Atmospheric and Earth System Research, University of Helsinki, Helsinki, FI-00014, Finland; 3 Air Pollution Management APM, Tampere, FI-33610, Finland; 4 Jayjock Associates, Langhorne, Pennsylvania, 19047, USA

**Keywords:** REACH, ECHA, Mandate, Exposure model criteria, Quantitative exposure, Qualitative exposure, Exposure modelling, Regulatory chemical safety decision-making

## Abstract

**Background:**

The European Chemicals Agency (ECHA) has been established to act as an independent body in the context of the implementation of the Regulation on the registration, evaluation, authorisation and restriction of chemicals (REACH) (Regulation (EC) 1907/2006). Quantitative exposure estimates are required for all exposure scenarios where hazardous emissions occur using exposure measurements or exposure models. REACH regulation specifies that exposure models need to be *appropriate* and *quantitative*. Here, we evaluated the criteria for regulatory exposure models by ECHA.

**Methods:**

The evaluation was performed by asking ECHA the criteria for exposure models.

**Results:**

ECHA does not specify any quality criteria for regulatory exposure models or have transparency requirements. Without quality criteria and transparency, there cannot be quality assurance or control. Thus, an *appropriate* model cannot be defined. ECHA does not recognize the *quantitative* term even though the fundamental requirement for quantitative exposure assessment is quantitative uncertainty assessment.

**Conclusions:**

As a result of these shortcomings, ECHA R.14 Guidance for occupational exposure assessment allows the use of non-physical models containing qualitative parameters based on non-accessible calibration databases and statistical evaluations. Because of the lack of transparency, non-physical model construct, and subjective input parameters, model results cannot be associated with real-world operational conditions, and quantitative uncertainty assessment is not feasible. This makes the models qualitative by definition and is not applicable to regulatory exposure modelling. This raises questions about whether ECHA has followed its regulatory mandates in implementing the REACH legislation.

## Plain language summary

ECHA has a mandate to implement REACH chemical regulation in the European Union. Quantitative exposure estimates are needed for hazardous chemicals' human health risk assessment. Quantitative exposure assessment can be conducted by using appropriate exposure models. However, ECHA does not provide quality criteria for regulatory exposure models in REACH, does not require transparency from the models applied in REACH, nor does it recognize the term quantitative. It is not feasible to conduct quality control or quality assurance without quality criteria or transparency requirements. As a result of these shortcomings, ECHA R.14 Guidance for occupational exposure assessment allows the use of non-physical models containing **qualitative** parameters based on non-accessible calibration databases and statistical evaluations. This raises logical questions about whether ECHA has followed its mandates in implementing the REACH legislation.

## Introduction

In registration, evaluation, authorisation and restriction of chemicals (REACH) regulation, exposure assessment shall be to make a quantitative or qualitative estimate of the dose/concentration of the substance to which humans and the environment are or may be exposed ( Regulation (EC) No 1907/2006 - Annex I §5.0). Exposure scenarios where hazardous emissions occur require quantitative exposure estimates to support the risk characterization ( ECHA, 2016).

Regulation (EC) No 1907/2006 - Annex I §5.2.1 specifies that “ *The exposure estimation entails three elements: (1) emission estimation; (2) assessment of chemical fate and pathways; and (3) estimation of exposure levels*”. The exposure estimates are associated with operational conditions and reported in the Chemical Safety Report. With this information, chemical users can ensure that exposure is adequately controlled under operational conditions specified in the Chemical Safety Report or conditions that favor lower exposure.

The exposure assessment can be conducted using measurements or exposure modelling. Annex I §5.2.5 specifies that “ *Where adequately measured representative exposure data are available, special consideration shall be given to them when conducting the exposure assessment. Appropriate models can be used for the estimation of exposure levels. Relevant monitoring data from substances with analogous use and exposure patterns or analogous properties can also be considered*”. Based on the Regulation (EC) No 1907/2006 - Annex I §5.0, the requirements for the exposure model are “ *appropriate*” and need to be “ *quantitative*” when hazardous emissions can occur.

The European Chemicals Agency (ECHA) mandates to implement Regulation (EC) No 1907/2006 are (adopted from https://echa.europa.eu/about-us/who-we-are/purpose):

1. Carry out technical, scientific, and administrative tasks related to the implementation of the EU’s chemicals legislation and policy.
2. Provide transparent, independent and high quality scientific opinions and decisions, which shall serve as the basis for the drafting and adoption of Union measures.
3. Collaborate and partner with EU bodies and Institutions, Member State authorities, as well as third countries and international organisations.
4. Provide tools, advice, and support to industry, with a particular focus on SMEs, in fulfilling their duties under chemical legislation.
5. Ensure that relevant, reliable, and objective information is available for the public and interested parties.

ECHA Chapter R.14 guidance specifies exposure models for occupational inhalation exposure assessments. The E-TEAM project evaluated parts of the generic inhalation exposure estimation tools classified as tier 1 tools, including Stoffenmanager (StM), which is currently called a tier 1.5 tool ( Lamb *et al.*, 2015). In ECHA Chapter R.14, the Advanced REACH Tool (ART) is similar to StM. ART is classified as a mechanistic exposure assessment tool classified as a tier 2 tool (note, ECHA does not follow the tier classification as internationally defined (e.g., Tielemans *et al.*, 2007); in ECHA, a lower tier model can produce lower exposure estimates than a higher tier model). During the past years, Koivisto *et al.* (Underlying data, section 1; ( Koivisto & Jayjock, 2025)) have found fundamental flaws in StM and ART models. Both StM and ART are based on calibration factors. The calibration databases are essential for model evaluation within the ISES Europe model evaluation group. However, calibration databases are not accessible from the model developers. Based on Regulation (EC) No 1049/2001 rule 6, these documents should be available for regulatory exposure models applied in REACH regulation.

In 2024, we requested ECHA access to the calibration databases, statistical evaluation methods, and the StM model evaluation report by the Dutch Ministry of Social Affairs and Employment. ECHA informed us that no document(s) that corresponded to the description given in our application were found. The response was that “ *According to Article 2(3) of Regulation (EC) No 1049/2001, the right of access, as defined in that Regulation, applies only to existing documents in possession of the Agency.*” The response raised questions about the currently used regulatory exposure models' transparency, quality criteria, assurance, and control. Such criteria are needed to establish an exposure model for regulatory chemical safety decision-making. This is one objective of the INTEGRANO (GA No. 101138414) project. The requirements were discussed with ECHA between 30 May 2024 and 28 Nov 2024. Here, we have summarized the findings according to our best understanding.

## ECHA communication

We sent five letters with questions about regulatory exposure model criteria, how the exposure models in ECHA R.14 guidance were evaluated, and how ECHA ensures that the exposure models comply with the “ *appropriate*” and “ *quantitative*” criteria. ECHA has not always provided a “yes/no” answer, and thus, the interpretation of the responses is not straightforward (Underlying data, section 2; Koivisto & Jayjock, 2025). Key questions, ECHA’s responses to these questions, and our interpretations are summarized in Table 1 (Underlying data). Unfortunately, clear answers were not provided for our questions in the last letter because ECHA considered those questions to be covered by the former letters.

## Results and discussion

ECHA does not specify any quality criteria for regulatory exposure models. Without quality criteria, there cannot be quality assurance or control. Thus, an “ *appropriate*” model cannot be defined. ECHA does not recognize the “ *quantitative*” term even though the “ *quantitative exposure model*” is described implicitly, for example, by WHO/IPCS (2005). We believe that if ECHA does not follow internationally recognized terminology, it should provide its own definition.

ECHA does not require transparency, which we see as violating the second mandate. Without transparency, the model construct cannot be evaluated, and the correct model application for the selected scenario cannot be ensured. For example, if model calibration databases are not available, it is impossible to ensure the database's quality or how the statistical evaluation is conducted. Transparency is a fundamental requirement for uncertainty analysis ( EFSA Scientific Committee, 2018a; EFSA Scientific Committee, 2018b; NRC, 2007; Simmonds *et al.*, 2022, p.; U.S. EPA, 2019; WHO/IPCS, 2005). For example, the U.S. Environmental Protection Agency (US-EPA) is responsible for ensuring that a model’s development and use are transparent and that models developed outside of the agency must meet the same acceptability and application criteria as models developed within the EPA ( NRC, 2007).

We agree that the model developers are responsible for building their models. Still, ECHA is responsible for providing acceptance criteria for the models (mandates 1 and 2) and ensuring that the models’ quality meets the requirements (mandate 5). Other regulatory bodies, such as the US-EPA or European Food Safety Authority (EFSA), have established such criteria and mandated that these criteria are complied ( EFSA Scientific Committee, 2018a; EFSA Scientific Committee, 2018b; NRC, 2007; U.S. EPA, 2019).

ECHA mentions that they can decide whether the assessments performed using models within the different processes are adequate. This decision addresses the model itself or the applicability of the model to the case. The decision appears to be subjective and arbitrary because it is unclear how such a decision can be made without quality criteria, transparency, or any defined understanding of what a “ *quantitative*” exposure model means.

A quantitative exposure model produces quantitative outputs using measurable variables, numerical inputs, and mathematical relationships based on physical and chemical laws ( NRC, 2007; WHO/IPCS, 2005). Quantitative exposure models provide predicted exposure profiles and associated uncertainties, allowing for variability and quantitative uncertainty analysis (e.g. Simmonds *et al.*, 2022). Uncertainty is related to the model parametrization and how well the model construct reflects the real environment. Models always have qualitative components related to their application in real-world systems. Qualitative components can be, e.g., impurities in processed materials, incomplete air mixing, assumptions related to worker behavior, or sinks for emissions other than ventilation exhaust air. Qualitative components are described in the modeling result as assumptions and boundary conditions that the user needs to comply with (e.g. Simmonds *et al.*, 2022). An *appropriate* exposure model considers all relevant exposure determinants so that qualitative components do not significantly impact exposure estimates and the result is sufficiently precise for safety decision-making. For example, a simple near-field/far-field exposure model predictive performance for solvents (non-spraying exposure scenarios) is within a factor of 0.3–3.7 times the measured concentrations with 93% of the values between 0.5 and 2 ( Abattan *et al.*, 2021). This accuracy is sufficient for regulatory chemical safety decision-making ( Jayjock *et al.*, 2011). However, reporting quality defines its final applicability for regulatory chemical decision-making.

ECHA R.14 recommends using models that do not have a physical construct, are based on unknown calibration factors based on exposure measurements in different exposure groups, and contain subjective parameters (e.g., 'high transfer speed' without specifying physical values or ranges) ( Koivisto *et al.*, 2022). Because there is no physical model construct and the model contains subjective input parameters, model results cannot be associated with real-world operational conditions. For example, a 'high transfer speed' of 1 kg/min can be a high transfer speed in pharmaceutical powder handling but a low in cement mixing. Due to the same reasons, the model uncertainties cannot be evaluated ( Hesse *et al.*, 2015; Koivisto *et al.*, 2022), and thus, the results cannot be called *quantitative* exposure estimates. Non-physical models recommended in ECHA R.14 do not require information on emission rates/factors. Emission estimation is one requirement in Regulation (EC) No 1907/2006 and it is unclear if the emission estimates are always reported in Chemical Safety Reports.

Such models can produce nearly any outcome, which can be seen in their predictive performance. For example, StM and ART predictability is in the range of 100 000 fold ( Lamb *et al.*, 2015) and 21 000 fold ( Lee, 2023), respectively. See Koivisto *et al.* (2022) for a more comprehensive list of performance studies. Spinazzè *et al.* (2017) showed how lower-tier models (ECETOC-TRA and StM) produced lower exposure estimates than higher-tier model ART. The reason for this is not known because of the model's characteristics; The magnitude and direction of the uncertainty introduced by the models cannot be assessed ( Hesse *et al.*, 2015; Koivisto *et al.*, 2022). Koivisto *et al.* (2022) stated that combining the model’s high uncertainties with subjective interpretation of exposure taxonomy, a tiered approach using, e.g., ECETOC-TRA, StM, and ART can produce nearly any outcome. Currently, such models are useful only to obtain compliance with REACH regulations, but they will not give information on workers' exposure and safety or provide helpful information on the effect of exposure determinants on workers' exposure levels.

ECHA does not provide numerical criteria for model performance. According to our understanding, other institutes, such as the US-EPA and EFSA, do not offer numerical criteria for exposure models. However, NRC (2007), a guidance document for US-EPA, specifies that if the model has been shown to produce erroneous results (false positives or false negatives), it should lead to a rejection of that model. ASTM D5157-19 provides numerical standards for indoor air quality models under well-specified conditions. A well-mixed model and a near-field/far-field model complied with the criteria by ASTM D5157-19 ( Arnold *et al.*, 2017).

ECHA has not formally evaluated the exposure models for compliance. The regulatory exposure models specified in ECHA R.14 were incorporated after expert discussions with as yet unidentified stakeholders. In fact, ECHA could not provide answers on who were the stakeholders in this process. They could not give the model quality criteria or how the evaluation was conducted. There appears to be no transparency criteria.

ECHA or the ECHA committees can reject an exposure model for regulatory exposure assessment. The rejection is usually based on undefined expert judgement applicable to the case at hand rather than explicit generic criteria. It is unclear how the evaluation is conducted because, according to ECHA, they do not formally evaluate the exposure models for compliance. Also, an assessment by ECHA using unspecified, generic criteria without explicitly defined quality criteria is not a technically rational approach, especially if the evaluation reports are not available for review.

ECHA can update its Guidance depending on the scientific outcomes from the International Society of Exposure Science (ISES) strategy 2020–2030 for exposure models. ECHA also monitors scientific development in international organizations such as the ISES group, the Society of Environmental Toxicology and Chemistry (SETAC), and the Organisation for Economic Co-operation and Development (OECD). However, ISES does not provide regulatory guidance or perform regulatory exposure model evaluations because they do not have a mandate. ECHA relies on “stakeholders” to implement the regulatory guidance, but ECHA’s contribution and responsibility are unclear. This is dramatically different from the practices of EFSA and US-EPA, which have established guidelines with quality criteria, have established a quality management system, and conduct self-reviews to improve their processes ( EFSA, 2024; NRC, 2007; U.S. EPA, 2019). Considering that we have found fundamental flaws in the highest tier models of the ECHA R.14 Guidance and that ECHA has not addressed those flaws for the past 6 years, we are not convinced that ECHA will ever actively develop its regulatory Guidance.

## Conclusions

ECHA has not developed quality criteria for regulatory exposure modelling, and thus, quality control is based on unspecified expert judgments. The details on which these judgments were made are unavailable to any independent scientific investigator. Indeed, ECHA does not require transparency from exposure models, which is in stark contrast to other chemical regulation bodies. Also, ECHA does not follow international terminology for exposure sciences, and thus, the interpretation of ECHA Guidance is not straightforward. These facts allow ECHA to recommend using qualitative exposure models in quantitative exposure assessment.

In contrast to the above ECHA practices, we decided in the INTEGRANO project to follow the US-EPA guidance for the regulatory exposure models, ensuring their global applicability.

We recommend that ECHA adopt the quality criteria, assurance, and control for regulatory exposure modelling from US-EPA and EFSA. Harmonization is a step towards globally accepted regulatory exposure assessment. Afterward, the ECHA Chapter R.14 exposure models should be objectively evaluated for compliance with these criteria. We see this as necessary to ensure the transparency of all models used for regulatory compliance.

## Ethics and consent

Ethical approval and consent were not required

## Institutional review board statement

Not applicable.

## Informed consent statement

Not applicable.

## Data Availability

The communication emails are available in Underlying data (anonymized).

## Data availability

### Underlying data

Zenodo: Underlying data for “How credible is REACH regulation without transparency, quality criteria, assurance, and control?” https://doi.org/10.5281/zenodo.15095459 ( Koivisto & Jayjock, 2025):

- Section 1, History of StM and ART evaluation
- Section 2, ECHA communication letters
- Section 3, ECHA review comments and responses
- Table 1, Answers extrapolated from ECHA communication Letters
