# Supplementary material for: How credible is REACH regulation without transparency, quality criteria, assurance, and control?
Source: Open Res Eur. 2025 Dec 21;5:100. Originally published 2025 Apr 10. [Version 3] doi: 10.12688/openreseurope.19510.3 (PMC12790599; doi:10.12688/openreseurope.19510.3)
Supplement: Peer review history

# Reviewer report: Reviewer response for version 3

**DOI:** 10.21956/openreseurope.24068.r66605

**Published:** 2026-01-10

**Authors:** Marlene Ågerstrand

**Affiliations:** 1 Stockholm University, Stockholm, Sweden

I am satisfied with the changes implemented by the authors. The revised manuscript effectively addresses a relevant topic that warrants increased scholarly attention, and I appreciate the authors’ efforts to highlight this issue for a wider readership.

Reviewer Expertise:

Chemical regulation

I confirm that I have read this submission and believe that I have an appropriate level of expertise to confirm that it is of an acceptable scientific standard.

# Reviewer report: Reviewer response for version 2

**DOI:** 10.21956/openreseurope.22833.r62116

**Published:** 2025-12-15

**Authors:** Peter Egeghy

**Affiliations:** 1 Center for Computational Toxicology and Exposure, Research Triangle Park, USA

The change of designation from "Research Article" to "Open Letter" has addressed and allayed my concerns.

Reviewer Expertise:

Exposure modeling

I confirm that I have read this submission and believe that I have an appropriate level of expertise to confirm that it is of an acceptable scientific standard.

# Reviewer report: Reviewer response for version 2

**DOI:** 10.21956/openreseurope.22833.r57503

**Published:** 2025-08-19

**Authors:** Marlene Ågerstrand

**Affiliations:** 1 Stockholm University, Stockholm, Sweden

The authors highlight a major concern: the lack of transparency in the calibration databases for two screening-level models. By pressing ECHA for greater openness, the authors provide an important public service. Robust and transparent exposure models are essential for managing chemical risks to health and the environment, while open data fosters innovation, supports academic research, and advances the development of safer, more sustainable chemical solutions.

 My concern with this paper is the categorisation as a “research article”. A research article is a detailed, structured report of an original scientific study. It typically presents a specific research question, explains the methods used to investigate it, presents data collected during the study, and analyses that data to draw a conclusion. A commentary, on the other hand, is a shorter, more opinion-driven piece that does not present original research data. Instead, it offers expert insight, interpretation, or critique on existing research, current trends, or issues in the field. Their purpose is often to stimulate discussion, guide interpretation, or provide context for ongoing debates.

 In the present case, the lack of a systematic approach to how the data have been analysed is making it difficult to categorise it as a research article. The authors could have used qualitative document analysis, which is a valid research approach involving systematic examination of texts to identify patterns, themes, and meanings. However, the paper fails to deliver on both the methodological rigour and performance expected for such a study. A clear description of the analysis process, particularly how the documents were examined, coded, and interpreted, is absent. This absence pushes the work closer to an informal commentary rather than a robust research article.

Reviewer Expertise:

Regulatory toxicology, EU chemical regulation

I confirm that I have read this submission and believe that I have an appropriate level of expertise to confirm that it is of an acceptable scientific standard, however I have significant reservations, as outlined above.

# Response

**Published:** 2025-08-19

**Authors:** Antti Joonas Koivisto

We thank the reviewer for valuable comments. These comments did not lead to changes, which are justified below. The authors highlight a major concern: the lack of transparency in the calibration databases for two screening-level models. By pressing ECHA for greater openness, the authors provide an important public service. Robust and transparent exposure models are essential for managing chemical risks to health and the environment, while open data fosters innovation, supports academic research, and advances the development of safer, more sustainable chemical solutions.

***Response:** Transparency is a fundamental requirement when applying modelling results to real-world systems. Underlying assumptions, model boundaries, and parameterisation must be reported so that the model result can be applied accordingly. However, this is only one primary concern regarding the exposure models given in ECHA R.14.*

*Other major concerns are that ECHA allows use of qualitative exposure models in regulatory exposure assessment under REACH and claims that the qualitative exposure models result in quantitative exposure estimates. Accuracy and uncertainty assessment are not possible for models without a physical construct (e.g. mass-balance), applying subjective parameters, and without transparency. Such models are qualitative models that are not applicable for tiered exposure modelling, such as screening the highest exposure potential under reasonable worst-case conditions. Stoffenmanager and the ART are not classified as screening-level models but quantitative exposure models for regulatory exposure assessment under the EU health and safety regulations Directive 98/24/EC—risks related to chemical agents at work and Directive 2004/37/EC—carcinogens or mutagens at work (Cherrie et al., 2020; Schlueter and Tischer, 2020; Schlüter et al., 2022; Schlüter and Spinazzè, 2023). For example, Stoffenmanager is claimed to be directly applicable to ( https://oshwiki.osha.europa.eu/en/themes/stoffenmanagerr-smart-chemicals-management-and-business-continuity; Accessed 19 Oct 2025):*

- *Directive 98/24/EC - risks related to chemical agents at work of 7 April 1998 on the protection of the health and safety of workers from the risks related to chemical agents at work (fourteenth individual Directive within the meaning of Article 16(1) of Directive 89/391/EEC)*
- *Directive 2004/37/EC - carcinogens or mutagens at work of 29 April 2004 on the protection of workers from the risks related to exposure to carcinogens or mutagens at work (Sixth individual Directive within the meaning of Article 16(1) Directive 89/391/EEC)*
- *Regulation (EC) No 1907/2006 - REACH of 18 December 2006 concerning the Registration, Evaluation, Authorisation and Restriction of Chemicals (REACH) and establishing a European Chemicals Agency. Occupational exposure assessment (Chapter R.14) (23/08/2016) of the REACH technical Guidance on Information Requirements and Chemical Safety Assessment*
- *Regulation (EC) No 1272/2008 - classification, labelling and packaging of substances and mixtures of the European Parliament and of the Council of 16 December 2008 on classification, labelling and packaging of substances and mixtures, amending and repealing Directives 67/548/EEC and 1999/45/EC, and amending Regulation (EC) No 1907/2006 (Text with EEA relevance)*

* Additional legislation (only latest list mentioned):*

- *Directive 91/322/EEC - indicative limit values of 29 May 1991 on establishing indicative limit values by implementing Council Directive 80/1107/EEC on the protection of workers from the risks related to exposure to chemical, physical and biological agents at work.*
- *Directive 2019/1831 - indicative occupational exposure limit values of 24 October 2019 establishing a fifth list of indicative occupational exposure limit values pursuant to Council Directive 98/24/EC and amending Commission Directive 2000/39/EC*

* The biological agents legislation directly applicable to Stoffenmanager® is:*

- *Directive 2000/54/EC - biological agents at work of the European Parliament and of the Council of 18 September 2000 on the protection of workers from risks related to exposure to biological agents at work (seventh individual directive within the meaning of Article 16(1) of Directive 89/391/EEC)*

My concern with this paper is the categorisation as a “research article”. A research article is a detailed, structured report of an original scientific study. It typically presents a specific research question, explains the methods used to investigate it, presents data collected during the study, and analyses that data to draw a conclusion. A commentary, on the other hand, is a shorter, more opinion-driven piece that does not present original research data. Instead, it offers expert insight, interpretation, or critique on existing research, current trends, or issues in the field. Their purpose is often to stimulate discussion, guide interpretation, or provide context for ongoing debates.

**Response:** *We agree. This article was supposed to be published as an Open letter and not a Research article. We ask the editor to change the article to “Open Letter” from “Research Article”. We repeat this request to the Editor.*

 In the present case, the lack of a systematic approach to how the data have been analysed is making it difficult to categorise it as a research article. The authors could have used qualitative document analysis, which is a valid research approach involving systematic examination of texts to identify patterns, themes, and meanings. However, the paper fails to deliver on both the methodological rigour and performance expected for such a study. A clear description of the analysis process, particularly how the documents were examined, coded, and interpreted, is absent. This absence pushes the work closer to an informal commentary rather than a robust research article.

**Response:** *This work focuses on the regulatory exposure model requirements specified by ECHA. Model evaluation was carried out for StM and ART in Koivisto et al. (2022, 2018), but this work was ignored. This is explained in the Underlying data "History of StM and ART evaluation". ECETOC-TRA, EMKG-EXPO-TOOL, MEASE, EASE, etc. models based on multipliers and subjective selections have a similar structure to "more sophisticated" StM and ART, which fail in quantitative exposure assessment. Thus, these models are qualitative as well. We also request that "the ECHA Chapter R.14 exposure models should be objectively evaluated for compliance with these criteria [referring to US-EPA and EFSA]." We wrote in the "History of StM and ART evaluation" that "E-TEAM reports have not evaluated the models’ theoretical backgrounds at the scientific level. There are no independent evaluation reports from StM and ART other than Koivisto et al. (2022, 2018) in which scientific issues were laid out but not responded to." We see that "...but not responded to" holds because Cherrie et al. (2020) and Fransman et al. (2022) did not address the scientific parts in their response letters. We extracted all the information related to exposure models criteria within the REACH regulation from ECHA and presented the results transparently. Feel free to propose a better approach which is more systematic. Terminology was discussed within ISES Europe, and this discussion is followed here. Unfortunately, the terminology study has not yet been published, but we can provide the proposals. However, that is irrelevant if ECHA uses its terminology without following international definitions or providing its own definitions. See also the comments for Peter Egeghy.   References Cherrie, J.W., Fransman, W., Heussen, G.A.H., Koppisch, D., Jensen, K.A., 2020. Exposure Models for REACH and Occupational Safety and Health Regulations. IJERPH 17, 383. https://doi.org/10.3390/ijerph17020383 Fransman, W., Arnone, M., Borghi, F., Cattaneo, A., Cavallo, D.M., Cherrie, J.W., Franken, R., Galea, K.S., van der Haar, R., Heussen, G.A.H., Jensen, K.A., Koponen, M., Koppisch, D., Kromhout, H., Luo, Y.-S., McNally, K., Säämänen, A., Spinazzè, A., van Tongeren, M., Vanoirbeek, J., Verpaele, S., Vetter, D., Viegas, S., Warren, N., 2022. Response Letter to Koivisto et al . ‘Evaluating the Theoretical Background of STOFFENMANAGER® and the Advanced REACH Tool.’ Annals of Work Exposures and Health 66, 543–549. https://doi.org/10.1093/annweh/wxac001 Koivisto, A.J., Jayjock, M., Hämeri, K.J., Kulmala, M., Van Sprang, P., Yu, M., Boor, B.E., Hussein, T., Koponen, I.K., Löndahl, J., Morawska, L., Little, J.C., Arnold, S., 2022. Evaluating the Theoretical Background of STOFFENMANAGER® and the Advanced REACH Tool. Annals of Work Exposures and Health 66, 520–536. https://doi.org/10.1093/annweh/wxab057 Koivisto, A.J., Jensen, A.C.Ø., Koponen, I.K., 2018. The general ventilation multipliers calculated by using a standard Near-Field/Far-Field model. Journal of Occupational and Environmental Hygiene 15, D38–D43. https://doi.org/10.1080/15459624.2018.1440084 Schlueter, U., Tischer, M., 2020. Validity of Tier 1 Modelling Tools and Impacts on Exposure Assessments within REACH Registrations—ETEAM Project, Validation Studies and Consequences. IJERPH 17, 4589. https://doi.org/10.3390/ijerph17124589 Schlüter, U., Arnold, S., Borghi, F., Cherrie, J., Fransman, W., Heussen, H., Jayjock, M., Jensen, K.A., Koivisto, J., Koppisch, D., Meyer, J., Spinazzè, A., Tanarro, C., Verpaele, S., Von Goetz, N., 2022. Theoretical Background of Occupational-Exposure Models—Report of an Expert Workshop of the ISES Europe Working Group “Exposure Models.” IJERPH 19, 1234. https://doi.org/10.3390/ijerph19031234 Schlüter, U., Spinazzè, A., 2023. Understanding the limitations and application of occupational exposure models in a REACH context. Journal of Occupational and Environmental Hygiene 20, 336–349. https://doi.org/10.1080/15459624.2023.2208188*

# Reviewer report: Reviewer response for version 1

**DOI:** 10.21956/openreseurope.21109.r55892

**Published:** 2025-07-25

**Authors:** Peter Egeghy

**Affiliations:** 1 Center for Computational Toxicology and Exposure, Research Triangle Park, USA

The article titled “How credible is REACH regulation without transparency, quality criteria, assurance, and control?” argues that ECHA may not be in compliance with its regulatory mandates for implementing the REACH legislation. This argument is based on (1) REACH Regulation (EC) No 1907/2006, Annex I, §5.0 requires quantitative exposure estimates to support the risk characterization, (2) ECHA allows the use of non-physical models containing qualitative parameters based on non-accessible calibration databases, and (3) the authors contend that “the fundamental requirement for quantitative exposure assessment is quantitative uncertainty assessment.” The article is well written, and both authors are well respected with strong reputations in the area of human exposure modeling.

 One of the main issues that the authors discuss is the lack of transparency that arises form the use of non-accessible calibration databases for two particular screening-level models: Advanced REACH Tool (ART) and Stoffenmanager (StM). Indeed, many regulatory organizations are prohibited, or at least highly discouraged, from using models that are not fully transparent with publicly accessible algorithms and training data. A lack of transparency undermines the legitimacy of any decisions that rely on those models. The authors are providing the public and other stakeholders a great service by pushing ECHA towards greater transparency and accountability.

 This article is important to the scientific community because robust exposure prediction models are critical to managing the human health and ecological risks posed by chemical substances. Moreover, open access to data encourages research and innovation within the chemical industry and academia. Full transparency allows researchers to build upon existing knowledge, leading to the development of safer and more sustainable chemical products and processes.

 The article is categorized as a “research article,” but despite following the standardized structure of a research article, it more closely resembles a commentary. The Methods of the investigation consisted of sending five letters to ECHA asking specific questions about criteria for use of the models for regulatory purposes (i.e., registration of chemical substances to ensure that the risks associated with their use are properly managed). While there is an abundance of published articles that evaluate available lower-tier models for occupational exposure [which can be accessed through Google Scholar with search term "‘evaluation’ AND (‘Advance Reach Tool’ OR ‘Stoffenmanager’ OR ‘Ecetoc TRA”"), the authors have not performed a comprehensive evaluation of those articles.

 It appears that many of the criticisms of in the current manuscript were previously published by the authors (Koivisto, Jayjock, Hämeri, et al., 2022 [Ref 1]) and that the criticisms were elaborately refuted by the developers of the models (Fransman, Arnone, Borghi, et al., 2022 [Ref 6]). While the authors of the current manuscript cite their previous article multiple times, they fail to acknowledge the rebuttal while repeating the criticisms.

 One of the key criticisms levied by the authors is that the (screening level) models accepted by ECHA fail to meet the definition of a “quantitative” exposure model. According to the authors a quantitative exposure model must not only rely on measurable inputs and mathematical relationships based on physical and chemical laws but must also allow for quantitative uncertainty analysis. The latter requirement does not appear to be aligned with internationally recognized terminology. For example, the seminal textbook by Rappaport and Kupper (2008)[Ref 5] makes no mention of a requirement for uncertainty analysis. Both ART and StM are referred to as “quantitative exposure models” in the scientific literature (e.g., Lee, Lee, and Kim, 2019 [Ref 4]). The citation provided by the authors of the current manuscript to support their contention of the fundamental requirement for quantitative uncertainty assessment (Simmonds EG, Adjei KP, Andersen CW, et al., 2022) acknowledges that quantification of model-related uncertainty is not consistent across scientific fields and presents a concept of uncertainty that is much more expansive than in the current manuscript. Likewise, Fryer et al. (2006) [Ref 2] describe four broad categories of uncertainty in exposure modeling, namely scenario uncertainty, model uncertainty, parameter variability, and parameter uncertainty.

 The authors seem to overlook the fit-for-purpose nature of lower-tier screening level models like ART and StM. The usefulness of these models lies in the ability to apply them in data-limited situations, such as the registration of new chemicals. For these models, accuracy is less important than producing conservative estimates to ensure health protection. As described in Schlüter et al. (2022) [Ref 3], higher-tier, mass balance-based models are able to provide a more refined quantitative estimate of exposure, but at a far higher cost (e.g., for input data) and far more limited applicability.

 Specific comments:

 Introduction, 1 ^st^ sentence: the phrase “shall be to make” is awkward. An awkward first sentence can bias a reader.

 Introduction, 2 ^nd^ paragraph: These three elements refer to exposure assessment across the lifecycle of a chemical whereas this manuscript is focused on occupational exposures.

 Introduction, 3 ^rd^ paragraph: The first four items of the bulleted list do not seem relevant to this manuscript.

 Introduction, 4th paragraph: The last sentence states that calibration databases are required to be available under REACH regulation. What fraction of available lower-tier models make the calibration database available?

 Introduction, 5th paragraph: Please explain why the authors relied on responses to questions from ECHA rather than on the multitude of REACH-related model evaluations available in the scientific literature.

 Results, 1 ^st^ paragraph: It is not clear what is meant by “ECHA does not recognize the ‘quantitative’ term”. Also why cite a source that only “implicitly” defines “quantitative exposure model” when there are numerous sources that explicitly define the term?

 Results, 5th paragraph: The definition of “quantitative exposure model” should come much earlier in the manuscript.

 Results, 5th paragraph: The paragraph is about uncertainty, yet the final two sentences refer to “accuracy,” which, as the authors know, is a related but different concept.

 Results, 6 ^th^ paragraph: The text presents an example of how a transfer speed can be high on one context yet low in another.  Perhaps it is this context-related relativity that forces the reliance on subjective input parameters in screening-level models.

 Results, 7 ^th^ paragraph: The last sentence states that models like StM and ART “will not give information on workers' exposure and safety or provide helpful information on the effect of exposure determinants on workers' exposure levels.” I do not think that is the purpose of these models and that it may not be fair to criticize a model (or category of models) for not being able to do something for which it is not intended.

 Results, 8 ^th^ paragraph: In the second sentence, I believe that he authors intended the affirmative (i.e., “do offer”) rather than the negative (“do not offer”). Otherwise this statement would contradict the last sentence of the third paragraph in the Results section.

 Results, last paragraph: Please provide a citation for the ISES strategy for exposure models.

 Disclaimer: *The views expressed in this peer review are those of the author and do not necessarily represent the views or the policies of the U.S. Environmental Protection Agency.*

Is the study design appropriate and does the work have academic merit?

Partly

Is the work clearly and accurately presented and does it cite the current literature?

Partly

If applicable, is the statistical analysis and its interpretation appropriate?

Not applicable

Are all the source data underlying the results available to ensure full reproducibility?

Yes

Are the conclusions drawn adequately supported by the results?

Partly

Are sufficient details of methods and analysis provided to allow replication by others?

Yes

Reviewer Expertise:

Exposure modeling

I confirm that I have read this submission and believe that I have an appropriate level of expertise to confirm that it is of an acceptable scientific standard, however I have significant reservations, as outlined above.

## References

[1] : Evaluating the Theoretical Background of STOFFENMANAGER® and the Advanced REACH Tool. *Annals of Work Exposures and Health* .2022;66(4) : 10.1093/annweh/wxab057 520-536 10.1093/annweh/wxab057 PMC903012434365499

[2] : Human exposure modelling for chemical risk assessment: a review of current approaches and research and policy implications. *Environmental Science & Policy* .2006;9(3) : 10.1016/j.envsci.2005.11.011 261-274 10.1016/j.envsci.2005.11.011

[3] : Theoretical Background of Occupational-Exposure Models—Report of an Expert Workshop of the ISES Europe Working Group “Exposure Models”. *International Journal of Environmental Research and Public Health* .2022;19(3) : 10.3390/ijerph19031234 10.3390/ijerph19031234 PMC883498835162257

[4] : Comparison of Quantitative Exposure Models for Occupational Exposure to Organic Solvents in Korea. *Annals of Work Exposures and Health* .2019;63(2) : 10.1093/annweh/wxy087 197-217 10.1093/annweh/wxy087 30445617

[5] : Quantitative Exposure Assessment.2008;

[6] : Response Letter to Koivistoet al . ‘Evaluating the Theoretical Background of STOFFENMANAGER® and the Advanced REACH Tool’. *Annals of Work Exposures and Health* .2022;66(4) : 10.1093/annweh/wxac001 543-549 10.1093/annweh/wxac001 35182067

# Response

**Published:** 2025-07-30

**Authors:** Antti Joonas Koivisto

We thank the reviewer for the valuable comments.

 The article is categorized as a “research article,” but despite following the standardized structure of a research article, it more closely resembles a commentary.

 With the Editor, we agreed to publish the manuscript as Open Letter rather than a Research Article.

 I sent a separate message to the Editor The Methods of the investigation consisted of sending five letters to ECHA asking specific questions about criteria for use of the models for regulatory purposes (i.e., registration of chemical substances to ensure that the risks associated with their use are properly managed). While there is an abundance of published articles that evaluate available lower-tier models for occupational exposure [which can be accessed through Google Scholar with search term "‘evaluation’ AND (‘Advance Reach Tool’ OR ‘Stoffenmanager’ OR ‘Ecetoc TRA”"), the authors have not performed a comprehensive evaluation of those articles. We agree, this is not related to the quality criteria for exposure models. Also, the Underlying Data, Section 1, “ *The history of StM and ART evaluation”*, reveals that there are no other evaluation studies than ours. See justification from Table R1. Table R1 can be added to the Underlying Data, Section 1, to support our statement. **Table R1.** STOFFENMANAGER ^®^ and ART evaluation studies. Developmental studies can be found from (Koivisto et al., 2022a). Last updated Dec 2024. **Study** **Investigation** Hesse et al. (2015) Theoretical evaluation of STOFFENMANAGER ^®^ concept Hesse et al. (2015) Uncertainty analysis of STOFFENMANAGER ^®^ algorithm and parameter Koivisto et al. (2018) Evaluation of the general ventilation multipliers Cherrie et al. (2020) Evaluation of the general ventilation multipliers Tischer et al. (2017) Commentary on ETEAM work Schlueter and Tischer (2020) Summary of the ETEAM work Fransman (2017) Editorial Schinkel et al. (2014) User reliability study. Koppisch et al. (2012) Comparison of tools estimates with exposure measurements Schinkel et al. (2010) Comparison of tools estimates with exposure measurements Hofstetter et al. (2013) Comparison of tools estimates with exposure measurements LeBlanc et al. (2018) Comparison of tools estimates with exposure measurements Jankowska et al. (2015) Comparison of tools estimates with exposure measurements Lamb et al. (2017) Comparison of tools estimates with exposure measurements Lamb et al. (2015) Comparison of tools estimates with exposure measurements Koivisto et al. (2015) Comparison of tools estimates with exposure measurements Landberg et al. (2015) Comparison of tools estimates with exposure measurements Landberg et al. (2018) Comparison of tools estimates with exposure measurements Landberg et al. (2019) Comparison of tools estimates with exposure measurements Landberg et al. (2017) Comparison of tools estimates with exposure measurements E. G. Lee et al. (2019) Comparison of tools estimates with exposure measurements Spinazzè et al. (2017) Comparison of tools estimates with exposure measurements Savic et al. (2017a) Comparison of tools estimates with exposure measurements E. G. Lee et al. (2019) Comparison of tools estimates with exposure measurements van Tongeren et al. (2017) Comparison of tools estimates with exposure measurements S. Lee et al. (2019) Comparison of tools estimates with exposure measurements Lee et al. (2020) STOFFENMANAGER ^®^ comparison study between communicated and calculated exposure estimates and development Riedmann et al. (2015) Sensitivity analysis Lee and Ceballos (2020) Adoption of exposure assessment tools to assist in providing respiratory protection recommendations Spinazzè et al. (2020) Comparison study between communicated and calculated exposure estimates Spinazzè et al. (2019) Performance in different exposure scenarios and between-user reliability Koivisto et al. (2019) Review of modeling methods Savic et al. (2018, 2017b, 2016) TREXMO Sailabaht et al. (2018) Welding fume extension Huang and Wu (2019) Occupational exposure assessment study (McNally, 2023) Comparison of tools estimates with exposure measurements (Jones, 2022) Personal opinion (Fransman et al., 2022) Limited response to Koivisto et al. (2022). Fransmann et al. (2022) did not respond to the Corrigendum letter by Koivisto et al. The Response letter was not accepted for publication by the Editor. (Cherrie, 2023) Commentary, Cherrie (2023) did not respond to the Corrigendum letter sent by Koivisto. (Lee, 2023) Comparison of tools estimates with exposure measurements (Sabic et al., 2024) Comparison of tools estimates with exposure measurements (Anhäuser et al., 2024) Comparison of tools estimates with exposure measurements (Jiang et al., 2024) Comparison of tools estimates with exposure measurements **STM development studies** Anhäuser, L., Piorr, B., Arnone, M., Wegscheider, W., Gerding, J., 2024. Occupational inhalation exposure during surface disinfection—exposure assessment based on exposure models compared with measurement data. J Expo Sci Environ Epidemiol 34, 345–355. https://doi.org/10.1038/s41370-023-00633-y Cherrie, J., Maccalman, L., Fransman, W., Tielemans, E., Tischer, M., Van Tongeren, M., 2011. Revisiting the Effect of Room Size and General Ventilation on the Relationship between Near- and Far-Field Air Concentrations. The Annals of Occupational Hygiene. https://doi.org/10.1093/annhyg/mer092 Cherrie, J.W., 2023. Occupational exposure science. Annals of Work Exposures and Health 67, 915–919. https://doi.org/10.1093/annweh/wxad052 Cherrie, J.W., 1999. The Effect of Room Size and General Ventilation on the Relationship Between Near and Far-Field Concentrations. Applied Occupational and Environmental Hygiene 14, 539–546. https://doi.org/10.1080/104732299302530 Cherrie, J.W., Fransman, W., Heussen, G.A.H., Koppisch, D., Jensen, K.A., 2020. Exposure Models for REACH and Occupational Safety and Health Regulations. IJERPH 17, 383. https://doi.org/10.3390/ijerph17020383 Fransman, W., 2017. How Accurate and Reliable Are Exposure Models? Annals of Work Exposures and Health 61, 907–910. https://doi.org/10.1093/annweh/wxx068 Fransman, W., Arnone, M., Borghi, F., Cattaneo, A., Cavallo, D.M., Cherrie, J.W., Franken, R., Galea, K.S., van der Haar, R., Heussen, G.A.H., Jensen, K.A., Koponen, M., Koppisch, D., Kromhout, H., Luo, Y.-S., McNally, K., Säämänen, A., Spinazzè, A., van Tongeren, M., Vanoirbeek, J., Verpaele, S., Vetter, D., Viegas, S., Warren, N., 2022. Response Letter to Koivisto *et al* . ‘Evaluating the Theoretical Background of STOFFENMANAGER® and the Advanced REACH Tool.’ Annals of Work Exposures and Health 66, 543–549. https://doi.org/10.1093/annweh/wxac001 Fransman, W., Cherrie, J., Van Tongeren, M., Schneider, T., Tischer, M., Schinkel, J., Marquart, H., warren, N., Spankie, S., Kromhout, H., Tielemans, E., 2013. Development of a mechanistic model for the Advanced REACH Tool (ART) -Version 1.5- (No. V9009). TNO. Fransman, W., Van Tongeren, M., Cherrie, J.W., Tischer, M., Schneider, T., Schinkel, J., Kromhout, H., Warren, N., Goede, H., Tielemans, E., 2011. Advanced Reach Tool (ART): development of the mechanistic model. Ann Occup Hyg 55, 957–979. https://doi.org/10.1093/annhyg/mer083 Hesse, S., Hanh, St., Schroeder, K., Mangelsdorf, I., Lamb, J., van Tongeren, M., 2015. Evaluation of Tier 1 Exposure Assessment Models under REACH (eteam) Project - Substudy Report on Uncertainty of Tier 1 Models (No. Project F 2303). BaUa, Dortmund/Berlin/Dresden 2015. Hesse, S, Schroeder, K., Mangelsdorf, I., Lamb, J., Van Tongeren, M., 2015. Evaluation of Tier 1 Exposure Assessment Models under REACH (eteam) Project, Substudy Report on Gathering of Background Information and Conceptual Evaluation. BaUa, Dortmund/Berlin/Dresden. Hofstetter, E., Spencer, J.W., Hiteshew, K., Coutu, M., Nealley, M., 2013. Evaluation of recommended REACH exposure modeling tools and near-field, far-field model in assessing occupational exposure to toluene from spray paint. Ann Occup Hyg 57, 210–220. https://doi.org/10.1093/annhyg/mes062 Huang, S., Wu, K., 2019. Health Risk Assessment of Photoresists Used in an Optoelectronic Semiconductor Factory. Risk Analysis 39, 2625–2639. https://doi.org/10.1111/risa.13366 Jankowska, A., Czerczak, S., Kucharska, M., Wesołowski, W., Maciaszek, P., Kupczewska-Dobecka, M., 2015. Application of predictive models for estimation of health care workers exposure to sevoflurane. Int J Occup Saf Ergon 21, 471–479. https://doi.org/10.1080/10803548.2015.1086183 Jiang, W., Wu, Z., Zhang, M., Zhang, H., 2024. Comparative Study of Exposure Assessment of Dust in Building Materials Enterprises Using ART and Monte Carlo. Safety and Health at Work 15, 33–41. https://doi.org/10.1016/j.shaw.2023.12.003 Jones, R.M., 2022. Models in Occupational Hygiene. Annals of Work Exposures and Health 66, 417–418. https://doi.org/10.1093/annweh/wxac016 Koivisto, A.J., Jayjock, M., Hämeri, K.J., Kulmala, M., Van Sprang, P., Yu, M., Boor, B.E., Hussein, T., Koponen, I.K., Löndahl, J., Morawska, L., Little, J.C., Arnold, S., 2022. Evaluating the Theoretical Background of STOFFENMANAGER® and the Advanced REACH Tool. Annals of Work Exposures and Health 66, 520–536. https://doi.org/10.1093/annweh/wxab057 Koivisto, A.J., Jensen, A.C.Ø., Koponen, I.K., 2018. The general ventilation multipliers calculated by using a standard Near-Field/Far-Field model. Journal of Occupational and Environmental Hygiene 15, D38–D43. https://doi.org/10.1080/15459624.2018.1440084 Koivisto, A.J., Jensen, A.C.Ø., Levin, M., Kling, K.I., Maso, M.D., Nielsen, S.H., Jensen, K.A., Koponen, I.K., 2015. Testing the near field/far field model performance for prediction of particulate matter emissions in a paint factory. Environ. Sci.: Processes Impacts 17, 62–73. https://doi.org/10.1039/C4EM00532E Koivisto, A.J., Kling, K.I., Hänninen, O., Jayjock, M., Löndahl, J., Wierzbicka, A., Fonseca, A.S., Uhrbrand, K., Boor, B.E., Jiménez, A.S., Hämeri, K., Maso, M.D., Arnold, S.F., Jensen, K.A., Viana, M., Morawska, L., Hussein, T., 2019. Source specific exposure and risk assessment for indoor aerosols. Science of The Total Environment 668, 13–24. https://doi.org/10.1016/j.scitotenv.2019.02.398 Koppisch, D., Schinkel, J., Gabriel, S., Fransman, W., Tielemans, E., 2012. Use of the MEGA Exposure Database for the Validation of the Stoffenmanager Model. The Annals of Occupational Hygiene 56, 426–439. https://doi.org/10.1093/annhyg/mer097 Lamb, J., Galea, K.S., Miller, B.G., Hesse, S., Van Tongeren, M., 2017. Between-User Reliability of Tier 1 Exposure Assessment Tools Used Under REACH. Annals of Work Exposures and Health 61, 939–953. https://doi.org/10.1093/annweh/wxx074 Lamb, J., Hesse, B., Miller, L., Maccalman, L., Schroeder, K., Cherrie, J., Van Tongeren, M., 2015. Evaluation of Tier 1 Exposure Assessment Models under REACH (eteam) Project  - Final Overall Project Summary Report. Landberg, H.E., Axmon, A., Westberg, H., Tinnerberg, H., 2017. A Study of the Validity of Two Exposure Assessment Tools: Stoffenmanager and the Advanced REACH Tool. Annals of Work Exposures and Health 61, 575–588. https://doi.org/10.1093/annweh/wxx008 Landberg, H.E., Berg, P., Andersson, L., Bergendorf, U., Karlsson, J.-E., Westberg, H., Tinnerberg, H., 2015. Comparison and Evaluation of Multiple Users’ Usage of the Exposure and Risk Tool: Stoffenmanager 5.1. ANNHYG 59, 821–835. https://doi.org/10.1093/annhyg/mev027 Landberg, H.E., Hedmer, M., Westberg, H., Tinnerberg, H., 2019. Evaluating the Risk Assessment Approach of the REACH Legislation: A Case Study. Annals of Work Exposures and Health 63, 68–76. https://doi.org/10.1093/annweh/wxy090 Landberg, H.E., Westberg, H., Tinnerberg, H., 2018. Evaluation of risk assessment approaches of occupational chemical exposures based on models in comparison with measurements. Safety Science 109, 412–420. https://doi.org/10.1016/j.ssci.2018.06.006 LeBlanc, M., Allen, J.G., Herrick, R.F., Stewart, J.H., 2018. Comparison of the near field/far field model and the advanced reach tool (ART) model V1.5: exposure estimates to benzene during parts washing with mineral spirits. International Journal of Hygiene and Environmental Health 221, 231–238. https://doi.org/10.1016/j.ijheh.2017.10.016 Lee, E.G., 2023. Evaluation of Stoffenmanager® and ART for Estimating Occupational Inhalation Exposures to Volatile Liquids. Annals of Work Exposures and Health 67, 402–413. https://doi.org/10.1093/annweh/wxac091 Lee, E.G., Ceballos, D.M., 2020. Adoption of Exposure Assessment Tools to Assist in Providing Respiratory Protection Recommendations. Annals of Work Exposures and Health 64, 547–557. https://doi.org/10.1093/annweh/wxaa023 Lee, E.G., Lamb, J., Savic, N., Basinas, I., Gasic, B., Jung, C., Kashon, M.L., Kim, J., Tischer, M., van Tongeren, M., Vernez, D., Harper, M., 2019. Evaluation of Exposure Assessment Tools under REACH: Part II-Higher Tier Tools. Ann Work Expo Health 63, 230–241. https://doi.org/10.1093/annweh/wxy098 Lee, S., Choi, S., Lee, K., 2020. Evaluation of Stoffenmanager and a New Exposure Model for Estimating Occupational Exposure to Styrene in the Fiberglass Reinforced Plastics Lamination Process. IJERPH 17, 4486. https://doi.org/10.3390/ijerph17124486 Lee, S., Lee, K., Kim, H., 2019. Comparison of Quantitative Exposure Models for Occupational Exposure to Organic Solvents in Korea. Annals of Work Exposures and Health 63, 197–217. https://doi.org/10.1093/annweh/wxy087 Marquart, H., Heussen, H., Le Deber, M., Noy, D., Tielemans, E., Schinkel, J., West, J., Van Der Schaaf, D., 2008. ‘Stoffenmanager’, a Web-Based Control Banding Tool Using an Exposure Process Model. The Annals of Occupational Hygiene. https://doi.org/10.1093/annhyg/men032 Marquart, H., Schneider, T., Goede, H., Tischer, M., Schinkel, J., Warren, N., Fransman, W., Spaan, S., Van Tongeren, M., Kromhout, H., Tielemans, E., Cherrie, J.W., 2011. Classification of occupational activities for assessment of inhalation exposure. Ann Occup Hyg 55, 989–1005. https://doi.org/10.1093/annhyg/mer072 McNally, K., 2023. How Valuable Are Small Measurement Datasets in Supplementing Occupational Exposure Models? A Numerical Study Using the Advanced Reach Tool. International Journal of Environmental Research and Public Health 20, 5386. https://doi.org/10.3390/ijerph20075386 Riedmann, R.A., Gasic, B., Vernez, D., 2015. Sensitivity Analysis, Dominant Factors, and Robustness of the ECETOC TRA v3, Stoffenmanager 4.5, and ART 1.5 Occupational Exposure Models: ECETOC TRA v3, Stoffenmanager 4.5, and ART 1.5 Occupational Exposure Models. Risk Analysis 35, 211–225. https://doi.org/10.1111/risa.12286 Sabic, S., Bell, D., Gasic, B., Schmid, K., Peter, T., Marcolli, C., 2024. Exposure assessment during paint spraying and drying using PTR-ToF-MS. Front. Public Health 11, 1327187. https://doi.org/10.3389/fpubh.2023.1327187 Sailabaht, A., Wang, F., Cherrie, J., 2018. Extension of the Advanced REACH Tool (ART) to Include Welding Fume Exposure. IJERPH 15, 2199. https://doi.org/10.3390/ijerph15102199 Savic, N., Gasic, B., Schinkel, J., Vernez, D., 2017a. Comparing the Advanced REACH Tool’s (ART) Estimates With Switzerland’s Occupational Exposure Data. Ann Work Expo Health 61, 954–964. https://doi.org/10.1093/annweh/wxx069 Savic, N., Gasic, B., Vernez, D., 2018. ART, Stoffenmanager, and TRA: A Systematic Comparison of Exposure Estimates Using the TREXMO Translation System. Annals of Work Exposures and Health 62, 72–87. https://doi.org/10.1093/annweh/wxx079 Savic, N., Gasic, B., Vernez, D., 2017b. Stoffenmanager® Algorithm Within Version 6 Differs from the Published Algorithm Within Old Versions and TREXMO. Ann Work Expo Health 61, 607–610. https://doi.org/10.1093/annweh/wxx026 Savic, N., Racordon, D., Buchs, D., Gasic, B., Vernez, D., 2016. TREXMO: A Translation Tool to Support the Use of Regulatory Occupational Exposure Models. ANNHYG 60, 991–1008. https://doi.org/10.1093/annhyg/mew042 Schinkel, J., Fransman, W., Heussen, H., Kromhout, H., Marquart, H., Tielemans, E., 2010. Cross-validation and refinement of the Stoffenmanager as a first tier exposure assessment tool for REACH. Occupational and Environmental Medicine 67, 125–132. https://doi.org/10.1136/oem.2008.045500 Schinkel, J., Fransman, W., McDonnell, P.E., Klein Entink, R., Tielemans, E., Kromhout, H., 2014. Reliability of the Advanced REACH Tool (ART). Ann Occup Hyg 58, 450–468. https://doi.org/10.1093/annhyg/met081 Schinkel, J., Warren, N., Fransman, W., van Tongeren, M., McDonnell, P., Voogd, E., Cherrie, J.W., Tischer, M., Kromhout, H., Tielemans, E., 2011. Advanced REACH Tool (ART): Calibration of the mechanistic model. J. Environ. Monit. 13, 1374. https://doi.org/10.1039/c1em00007a Schlueter, U., Tischer, M., 2020. Validity of Tier 1 Modelling Tools and Impacts on Exposure Assessments within REACH Registrations—ETEAM Project, Validation Studies and Consequences. IJERPH 17, 4589. https://doi.org/10.3390/ijerph17124589 Spinazzè, A., Borghi, F., Campagnolo, D., Rovelli, S., Keller, M., Fanti, G., Cattaneo, A., Cavallo, D.M., 2019. How to Obtain a Reliable Estimate of Occupational Exposure? Review and Discussion of Models’ Reliability. Int J Environ Res Public Health 16. https://doi.org/10.3390/ijerph16152764 Spinazzè, A., Borghi, F., Magni, D., Rovida, C., Locatelli, M., Cattaneo, A., Cavallo, D.M., 2020. Comparison between Communicated and Calculated Exposure Estimates Obtained through Three Modeling Tools. IJERPH 17, 4175. https://doi.org/10.3390/ijerph17114175 Spinazzè, A., Lunghini, F., Campagnolo, D., Rovelli, S., Locatelli, M., Cattaneo, A., Cavallo, D.M., 2017. Accuracy Evaluation of Three Modelling Tools for Occupational Exposure Assessment. Annals of Work Exposures and Health 61, 284–298. https://doi.org/10.1093/annweh/wxx004 Tielemans, E., Noy, D., Schinkel, J., Heussen, H., Van Der Schaaf, H., West, J., Fransman, W., 2008a. Stoffenmanager Exposure Model: Development of a Quantitative Algorithm. The Annals of Occupational Hygiene. https://doi.org/10.1093/annhyg/men033 Tielemans, E., Schneider, T., Goede, H., Tischer, M., Warren, N., Kromhout, H., Van Tongeren, M., Van Hemmen, J., Cherrie, J.W., 2008b. Conceptual model for assessment of inhalation exposure: defining modifying factors. Ann Occup Hyg 52, 577–586. https://doi.org/10.1093/annhyg/men059 Tischer, M., Lamb, J., Hesse, S., van Tongeren, M., 2017. Evaluation of Tier One Exposure Assessment Models (ETEAM): Project Overview and Methods. Annals of Work Exposures and Health 61, 911–920. https://doi.org/10.1093/annweh/wxx066 van Tongeren, M., Lamb, J., Cherrie, J.W., MacCalman, L., Basinas, I., Hesse, S., 2017. Validation of Lower Tier Exposure Tools Used for REACH: Comparison of Tools Estimates With Available Exposure Measurements. Annals of Work Exposures and Health 61, 921–938. https://doi.org/10.1093/annweh/wxx056

 It appears that many of the criticisms of in the current manuscript were previously published by the authors (Koivisto, Jayjock, Hämeri, et al., 2022 [Ref 1]) and that the criticisms were elaborately refuted by the developers of the models (Fransman, Arnone, Borghi, et al., 2022 [Ref 6]). While the authors of the current manuscript cite their previous article multiple times, they fail to acknowledge the rebuttal while repeating the criticisms. We have explained this thoroughly in Section 1 of the Underlying Data. We add the reference.

 One of the key criticisms levied by the authors is that the (screening level) models accepted by ECHA fail to meet the definition of a “quantitative” exposure model. According to the authors a quantitative exposure model must not only rely on measurable inputs and mathematical relationships based on physical and chemical laws but must also allow for quantitative uncertainty analysis. The latter requirement does not appear to be aligned with internationally recognized terminology. For example, the seminal textbook by Rappaport and Kupper (2008)[Ref 5] makes no mention of a requirement for uncertainty analysis. The latter requirement is an outcome of the first requirement; i.e., quantitative uncertainty assessment is possible for physical models, and their boundaries can be assessed. In the ISES Europe, we made a systematic evaluation of exposure terminology. The ISES Europe proposal was that “A quantitative exposure model is a mathematical representation that estimates the magnitude, frequency, duration, and route of exposure to a chemical. It combines information on the frequency, mode, and magnitude of contact with contaminated media to yield a quantitative value of exposure associated with quantitative uncertainty.“ We could not have access to Rappaport and Kupper. Both ART and StM are referred to as “quantitative exposure models” in the scientific literature (e.g., Lee, Lee, and Kim, 2019 [Ref 4]). Yes, as explained by Koivisto et al. (2022) StM and ART are called by different names without justification (See e.g. Table 1 in Koivisto et al. 2022).   The citation provided by the authors of the current manuscript to support their contention of the fundamental requirement for quantitative uncertainty assessment (Simmonds EG, Adjei KP, Andersen CW, et al., 2022) acknowledges that quantification of model-related uncertainty is not consistent across scientific fields and presents a concept of uncertainty that is much more expansive than in the current manuscript. Likewise, Fryer et al. (2006) [Ref 2] describe four broad categories of uncertainty in exposure modeling, namely scenario uncertainty, model uncertainty, parameter variability, and parameter uncertainty. We agree. We added Fryer et al. (2006) reference.

 The authors seem to overlook the fit-for-purpose nature of lower-tier screening level models like ART and StM. The usefulness of these models lies in the ability to apply them in data-limited situations, such as the registration of new chemicals. For these models, accuracy is less important than producing conservative estimates to ensure health protection. StM and ART are classified as higher-tier models, not screening-level models. One criterion for an adequate exposure model, as established by the Dutch Social Economic Council (Koivisto et al., 2022) and the OECD (ENV/CBC/MONO(2021)28), is that ≤10% of the modelled exposure estimates can exceed the measured exposure level. This criteria is not complied by StM or ART for which the the modelled 75th/90th percentile of the exposure distribution falls below the measured exposure level in >10% of the cases (e.g. Lamb et al. (2015); Riedmann et al. (2015); Landberg et al. (2017); Landberg et al. (2018); van Tongeren et al. (2017); Lee et al. (2018); Lee et al. (2020); Lee et al. (2023); Spinazzè et al. (2017). Similarly, Tier 1 models are shown to fails this criterion (e.g. Noij et al. (2023;  Tijdschrift voor toegepaste Arbowetenschap 2023;36(3)); Savic et al. (2023; 10.1093/annweh/wxad001); Spinazzè et al. (2017); van Tongeren et al. (2017); Landberg et al. (2018); Riedmann et al. (2015); Lamb et al. (2015)). StM or ART does not comply with the ASTM D5157-97 numerical criteria (we did not confirm this for Tier 1 models) (Koivisto et al. 2022). It is a misleading statement that these models would produce conservative estimates if they do not meet the minimum numerical criteria for an exposure model. This was also addressed in Koivisto et al. (2022), which Fransman et al. (2022) did not deny. This demonstrates how vital quality criteria is for exposure and risk communication. As described in Schlüter et al. (2022) [Ref 3], higher-tier, mass balance-based models are able to provide a more refined quantitative estimate of exposure, but at a far higher cost (e.g., for input data) and far more limited applicability. Emission is the point of departure for exposure and must be reported in the chemical safety report (REACH regulation - Annex I §5.2.1). Emission is a minimum requirement for predictive exposure assessment under various conditions and establishing conditions of use for chemical manufacturing and use (see e.g., https://doi.org/10.12688/openreseurope.19498.1; https://doi.org/10.12688/openreseurope.19658.2). Suppose the emission assessment is considered too high cost, and ECHA allows the use of non-physical models that do not require information on emissions. In that case, it is recommended to use these user-friendly models. What is a more refined quantitative estimate of exposure?

 Specific comments:

 Introduction, 1 ^st^ sentence: the phrase “shall be to make” is awkward. An awkward first sentence can bias a reader. The sentence was revised.

 Introduction, 2 ^nd^ paragraph: These three elements refer to exposure assessment across the lifecycle of a chemical whereas this manuscript is focused on occupational exposures. Yes, this is correct. No changes were made.

 Introduction, 3 ^rd^ paragraph: The first four items of the bulleted list do not seem relevant to this manuscript. We refer to these mandates in the main text. No changes were made. Introduction, 4th paragraph: The last sentence states that calibration databases are required to be available under REACH regulation. What fraction of available lower-tier models make the calibration database available? To the best of our knowledge, none of the calibration databases is currently available. No changes were made.

 Introduction, 5th paragraph: Please explain why the authors relied on responses to questions from ECHA rather than on the multitude of REACH-related model evaluations available in the scientific literature. The objective was to understand the criteria by ECHA for exposure models applied in the REACH regulation. Please see more from the Underlying Data section 1, which discusses the objective model evaluation, and Table R1. No changes were made.

 Results, 1 ^st^ paragraph: It is not clear what is meant by “ECHA does not recognize the ‘quantitative’ term”. Also why cite a source that only “implicitly” defines “quantitative exposure model” when there are numerous sources that explicitly define the term? ECHA considers a model result quantitative if the units are correct physical dimensions. Similarly, Fransman et al. (2022) state that the model is quantitative if it produces correct dimensions (here: mg/m3). This is not a sufficient criterion as explained in the main text and Underlying Data, Section 3, ECHA review comments and responses. We would be grateful if you could provide an explicit definition for “quantitative exposure model”. We added a citation to Underlying data, section 2; Koivisto & Jayjock, 2025).

 Results, 5th paragraph: The definition of “quantitative exposure model” should come much earlier in the manuscript. We agree, we moved this to the second paragraph in Results and discussion.

 Results, 5th paragraph: The paragraph is about uncertainty, yet the final two sentences refer to “accuracy,” which, as the authors know, is a related but different concept. We agree. We added “Model accuracy and uncertainty are related to the model parametrization and how well the model construct reflects the real environment (e.g., Fryer et al., 2006).”

 Results, 6 ^th^ paragraph: The text presents an example of how a transfer speed can be high on one context yet low in another.  Perhaps it is this context-related relativity that forces the reliance on subjective input parameters in screening-level models. See Marquart et al. (2008) for context-related relativity. No changes were made.

 Results, 7 ^th^ paragraph: The last sentence states that models like StM and ART “will not give information on workers' exposure and safety or provide helpful information on the effect of exposure determinants on workers' exposure levels.” I do not think that is the purpose of these models and that it may not be fair to criticize a model (or category of models) for not being able to do something for which it is not intended. This is the purpose of StM and ART (See e.g. Schlüter et al. 2022; https://doi.org/10.3390/ijerph19031234). They state that “ *STOFFENMANAGER® and ART are well-established and accepted tools that are used for regulatory risk assessments, e.g., for REACH or in the context of the EU occupational health and safety directive 98/24/EC (ref to https://doi.org/10.3390/ijerph17020383)*”. No changes were made.

 Results, 8 ^th^ paragraph: In the second sentence, I believe that the authors intended the affirmative (i.e., “do offer”) rather than the negative (“do not offer”). Otherwise this statement would contradict the last sentence of the third paragraph in the Results section. Negate is correct. We clarified the sentence as “According to our understanding, neither other institutes, such as the US-EPA and EFSA, offer numerical criteria for exposure models.” This does not contradict the third paragraph in the Results section because there are other criteria related to uncertainty assessment. If the model result, including uncertainty, yields compliance, then the result can be considered sufficiently accurate.

 Results, last paragraph: Please provide a citation for the ISES strategy for exposure models. This was added.

# Reviewer report: Reviewer response for version 1

**DOI:** 10.21956/openreseurope.21109.r55395

**Published:** 2025-07-25

**Authors:** Marlene Ågerstrand

**Affiliations:** 1 Stockholm University, Stockholm, Sweden

The paper critically examines the credibility of the EU’s REACH regulation for chemical safety, particularly occupational exposure modelling, arguing that it lacks sufficient transparency, quality criteria, assurance, and control mechanisms. The authors build their case by analyzing data access issues, inconsistencies in regulatory practices, and weaknesses in currently used exposure tools. The authors address a timely and under explored issue within EU chemical regulation, and with their work, they could contribute to improved policy accountability and scientific robustness.

 I have some suggestions for how the paper could be improved:

 While I sympathise with the author's frustration, they still need to maintain professional and objective language. As it is now, the paper borders on advocacy, which is rarely an advantage in such cases. There is also passive voice that needs to be removed.

 Consider making some language edits to improve clarity and precision, especially in the results and discussion sections. Some sections are wordy and repetitive rather than providing a logical order to the text.

 The first sentence of the introduction is missing a word: 'performed'?

 The paper lacks a systematic methodology section, so the text reads more like an anecdotal regulatory critique than a structured evaluation. A clear method section is important if you want to provide an unbiased critique and address institutional failure.

 Different abbreviations appear in the text without explanation and, in some cases, without justification, for example: ISES Europe model, INTEGRANO and E-TEAM.

 The statement is not sufficiently clarified, nor is the process by which the authors arrived at it: 'ECHA does not recognise the "quantitative" term...'

 The text would benefit from presenting counterarguments to balance the critique.

 Conclude the paper by listing your recommendations for ECHA and the EC.

 There are also places where modifiers are misplaced, for example:

 'Such models can produce nearly any outcome...' Which model is being referred to here?

 'There appears to be no transparency criteria.' What lacks transparency criteria?

 'This is one objective of INTEGRANO.' The preceding sentence reads, 'Such criteria are needed to establish an exposure model for regulatory chemical safety decision-making', and I assume this is not Integrano's objective.

Is the study design appropriate and does the work have academic merit?

Yes

Is the work clearly and accurately presented and does it cite the current literature?

No

If applicable, is the statistical analysis and its interpretation appropriate?

Not applicable

Are all the source data underlying the results available to ensure full reproducibility?

Yes

Are the conclusions drawn adequately supported by the results?

Partly

Are sufficient details of methods and analysis provided to allow replication by others?

No

Reviewer Expertise:

Regulatory toxicology, EU chemical regulation

I confirm that I have read this submission and believe that I have an appropriate level of expertise to state that I do not consider it to be of an acceptable scientific standard, for reasons outlined above.

# Response

**Published:** 2025-07-30

**Authors:** Antti Joonas Koivisto

The paper critically examines the credibility of the EU’s REACH regulation for chemical safety, particularly occupational exposure modelling, arguing that it lacks sufficient transparency, quality criteria, assurance, and control mechanisms. The authors build their case by analyzing data access issues, inconsistencies in regulatory practices, and weaknesses in currently used exposure tools. The authors address a timely and under explored issue within EU chemical regulation, and with their work, they could contribute to improved policy accountability and scientific robustness. We thank the reviewer for the valuable comments.

 I have some suggestions for how the paper could be improved:

 While I sympathise with the author's frustration, they still need to maintain professional and objective language. As it is now, the paper borders on advocacy, which is rarely an advantage in such cases. There is also passive voice that needs to be removed.

**Response: **We aim to explain the situation as clearly as possible to make an objective picture of the current situation in ECHA. In a previous study by Schlüter et al. 2022 (DOI: https://doi.org/10.3390/ijerph19031234), we balanced the text, which led to misinterpretations by other authors and ECHA. Removing passive voice can lead to misinterpreting the message of this manuscript. Unfortunately, we prefer to give as realistic a description of the situation as possible, but feel free to propose changes in passive sentences so that the message remains transparent and objective.

 Consider making some language edits to improve clarity and precision, especially in the results and discussion sections. Some sections are wordy and repetitive rather than providing a logical order to the text.

**Response: **We moved the description of the quantitative model to the second paragraph.

 The first sentence of the introduction is missing a word: 'performed'? The sentence was revised.

 The paper lacks a systematic methodology section, so the text reads more like an anecdotal regulatory critique than a structured evaluation. A clear method section is important if you want to provide an unbiased critique and address institutional failure.

**Response:** This article was supposed to be published as an Open letter and not a Research article. We ask the editor about this. I’m not sure what a better approach is than having a transparent discussion with ECHA. Terminology was discussed within ISES Europe, and this discussion is followed here. Unfortunately, the terminology study has not yet been published, but we can provide the proposals.

 Different abbreviations appear in the text without explanation and, in some cases, without justification, for example: ISES Europe model, INTEGRANO and E-TEAM.

**Response: **We agree. We had an abbreviation table, but it was removed from the online version. This was added once more. We were unable to identify abbreviations without justification. For example, “ *The calibration databases are essential for model evaluation within the ISES Europe model evaluation group.*” Should justify the use of the ISES Europe model evaluation group.

 The statement is not sufficiently clarified, nor is the process by which the authors arrived at it: 'ECHA does not recognise the "quantitative" term...'

**Response: **ECHA did not answer this. ECHA mentioned that “It is worth noting that a significant portion of the European scientific community has identified ART and Stoffenmanager as quantitative exposure models (Fransman et al., 2022).” (Underlying data, section 3). However, Fransman et al. (2022) state that the model is quantitative if it produces correct dimensions. This is not a sufficient criterion for quantitative assessment. No changes were made.

 The text would benefit from presenting counterarguments to balance the critique.

**Response: **We can add, for example, that if one divides the postal code by the shoe number and multiplies it by the occupational exposure limit value, it does not produce a quantitative exposure estimate, regardless of whether the dimensions are correct. However, this type of argument is not very constructive, because it should be clear to the scientific community. No changes were made.

 Conclude the paper by listing your recommendations for ECHA and the EC.

**Response: **This has been listed in the conclusions as “ *We recommend that ECHA adopt the quality criteria, assurance, and control for regulatory exposure modelling from US-EPA and EFSA. Harmonization is a step towards globally accepted regulatory exposure assessment. Afterward, the ECHA Chapter R.14 exposure models should be objectively evaluated for compliance with these criteria. We see this as necessary to ensure the transparency of all models used for regulatory compliance.*” No changes were made.

 There are also places where modifiers are misplaced, for example:

 'Such models can produce nearly any outcome...' Which model is being referred to here?

**Response: **We clarified that such models are subjective models.

 'There appears to be no transparency criteria.' What lacks transparency criteria?

**Response: **Calibration databases, their calculation methods, and the StM evaluation report. These were listed in the Introduction. No changes were made.

 'This is one objective of INTEGRANO.' The preceding sentence reads, 'Such criteria are needed to establish an exposure model for regulatory chemical safety decision-making', and I assume this is not Integrano's objective.

**Response: **INTEGRANO’s objective is to establish a mechanistic exposure model applicable for regulatory chemical safety decision-making in indoor environments. For that, we need to know what criteria have been established for exposure models, because a regulation that can be conducted by using arbitrary methods is not useful. No changes were made.
